# Rapamycin attenuates hypoxia-induced pulmonary vascular remodeling and right ventricular hypertrophy in mice

**DOI:** 10.1186/1465-9921-8-15

**Published:** 2007-02-24

**Authors:** Renate Paddenberg, Philipp Stieger, Anna-Laura von Lilien, Petra Faulhammer, Anna Goldenberg, Harald H Tillmanns, Wolfgang Kummer, Ruediger C Braun-Dullaeus

**Affiliations:** 1 Institute of Anatomy and Cell Biology, Giessen University, Giessen, Germany; 2 Department of Internal Medicine/Cardiology Giessen University, Giessen, Germany; 3 Department of Internal Medicine/Cardiology, Dresden University of Technology, Dresden, Germany

## Abstract

**Background:**

Chronic hypoxia induces pulmonary arterial hypertension (PAH). Smooth muscle cell (SMC) proliferation and hypertrophy are important contributors to the remodeling that occurs in chronic hypoxic pulmonary vasculature. We hypothesized that rapamycin (RAPA), a potent cell cycle inhibitor, prevents pulmonary hypertension in chronic hypoxic mice.

**Methods:**

Mice were held either at normoxia (N; 21% O_2_) or at hypobaric hypoxia (H; 0.5 atm; ~10% O_2_). RAPA-treated animals (3 mg/kg*d, i.p.) were compared to animals injected with vehicle alone. Proliferative activity within the pulmonary arteries was quantified by staining for Ki67 (positive nuclei/vessel) and media area was quantified by computer-aided planimetry after immune-labeling for α-smooth muscle actin (pixel/vessel). The ratio of right ventricle to left ventricle plus septum (RV/[LV+S]) was used to determine right ventricular hypertrophy.

**Results:**

Proliferative activity increased by 34% at day 4 in mice held under H (median: 0.38) compared to N (median: 0.28, p = 0.028) which was completely blocked by RAPA (median HO+RAPA: 0.23, p = 0.003). H-induced proliferation had leveled off within 3 weeks. At this time point media area had, however, increased by 53% from 91 (N) to 139 (H, p < 0.001) which was prevented by RAPA (H+RAPA: 102; p < 0.001). RV/[LV+S] ratio which had risen from 0.17 (N) to 0.26 (H, p < 0.001) was attenuated in the H+RAPA group (0.22, p = 0.041). For a therapeutic approach animals were exposed to H for 21 days followed by 21 days in H ± RAPA. Forty two days of H resulted in a media area of 129 (N: 83) which was significantly attenuated in RAPA-treated mice (H+RAPA: 92). RV/[LV+S] ratios supported prevention of PH (N 0.13; H 0.27; H+RAPA 0.17). RAPA treatment of N mice did not influence any parameter examined.

**Conclusion:**

Therapy with rapamycin may represent a new strategy for the treatment of pulmonary hypertension.

## Background

Pulmonary arterial hypertension (PAH), a disease of the small pulmonary arteries, is characterized by vascular proliferation and remodeling [1]. It results in a progressive increase in pulmonary vascular resistance and, ultimately, right ventricular failure and death. One trigger of PAH is hypoxia which acutely causes a rise in pulmonary blood pressure by vasoconstriction but chronically results in the structural remodeling of the pulmonary vasculature [2]. Medial thickening of small pulmonary arteries has long been recognized as one of the earliest pathologic features, indicating proliferation of smooth muscle cells (SMC) [3]. Indeed, smooth muscle cell proliferation in small, peripheral, normally nonmuscular pulmonary arterioles is a hallmark of PAH [4,5].

The current medical management of PAH is directed at vasodilatation rather than towards inhibition of smooth muscle cell proliferation [1]. However, recently an exciting new therapeutic avenue has been taken using a platelet-derived growth factor (PDGF) receptor antagonist to treat PAH in hypoxic rats [6]. This approach has even successfully been used in a single patient with end stage primary pulmonary hypertension [7]. Anti-proliferative therapy seems to offer a novel approach for treatment of PAH.

Rapamycin (sirolimus) is another very potent anti-proliferative drug. Through inhibition of its target, the mammalian Target of Rapamycin (mTOR), rapamycin blocks mitogen-induced signaling via phosphoinositide 3-kinase (PI3K) and protein kinase B (Akt) towards the cell cycle machinery in SMC in vitro and in vivo [8]. In cardiovascular medicine, rapamycin is successfully used as stent-coating for prevention of in-stent restenosis [9-11]. However, rapamycin also abrogates hypoxia-induced increase in proliferation of cultured smooth muscle and endothelial cells [12]. Furthermore, the requirement of PI3K, Akt, and mTOR in hypoxia-induced pulmonary artery adventitial fibroblast proliferation has been demonstrated recently [13].

On this background we hypothesized that rapamycin prevents and reverses hypoxia-induced vascular remodeling. Mice were injected with rapamycin or with vehicle alone (0.2% carboxymethylcellulose) and held either at normoxia (21% O_2_) or at hypobaric hypoxia (0.5 atm; ~10% O_2_). Frozen lung sections of mice kept for four days or three weeks at normoxia or hypobaric hypoxia were employed for double labeling for Ki67 (proliferating cells) and α-smooth muscle actin to quantify the proliferative activity of the pulmonary vasculature and to determine the vessel media area by computer-aided planimetry. In hematoxylin-eosin stained cross sections of frozen hearts, calculation of the ratio of the areas of right ventricular wall/[left ventricular wall + septum] and measurement of the diameters of individual cardiomyocytes served for the estimation of right ventricular hypertrophy. Our results demonstrated that rapamycin is able to attenuate hypoxia-induced proliferation and thickening of the pulmonary vasculature as well as right ventricular hypertrophy thereby supporting that anti-proliferative regimens offer a novel approach for anti-remodeling therapy in hypoxia-induced PAH.

## Methods

### Chemicals and antibodies

Rapamycin was a kind gift from Wyeth Pharmaceuticals (Muenster, Germany). FITC-conjugated monoclonal anti-α-smooth muscle actin antibody (clone 1A4) and 4',6-diamidino-2-phenyl-inodole (DAPI) were obtained from Sigma-Aldrich (Deisenhofen, Germany), rabbit polyclonal anti-Ki67 antibody from Novocastra Laboratories Ltd. (Dossenheim, Germany) and Cy3-conjugated donkey anti-rabbit antibody from Dianova (Hamburg, Germany).

### Animals and experimental protocol

FVB mice of both gender were obtained from Harlan Winkelmann (Paderborn, Germany) and used at 6–8 weeks of age. The animals were fed standard mouse chow and were allowed to take food and water ad libidum. All experiments conformed to the NIH guidelines to the care and use of experimental animals, and were approved by the local authorities.

The kinetic of proliferation within the walls of intrapulmonary vessels in response to reduced oxygen supply was examined in mice kept for 2, 3, 4, 10, 16, or 21 days in a hypobaric chamber. An air intake valve was adjusted to maintain intrachamber pressure at 380 mmHg (0.5 atm) while allowing adequate airflow through the chamber to prevent accumulation of CO_2 _and NH_3_. Control mice were kept at normobaric pressure (760 mmHg) at room air.

To examine the effect of rapamycin on hypoxia-induced vascular remodeling and right ventricular hypertrophy, age-matched mice were divided into 6 experimental groups: 1. untreated normoxic mice, 2. vehicle-treated normoxic mice, 3. rapamycin-treated normoxic mice, 4. untreated hypobaric mice, 5. vehicle-treated hypobaric mice, and 6. rapamycin-treated hypobaric mice. In some experiments solely four groups (vehicle-/rapamycin-treated mice at normoxia/hypoxia) were formed. For application of rapamycin or vehicle, the chamber was opened daily and the mice were weighed. An 1.75 mg/ml stock solution of rapamycin was freshly prepared every second day by homogenization of the drug in 0.2% carboxymethylcellulose as vehicle. Rapamycin was injected i.p. at 3 mg/kg*d in a final volume of 100 μl. Control mice received either the same volume of the vehicle or remained untreated.

### Tissue preparation

Mice were sacrificed by cervical dislocation and exsanguinated by cutting the vena cava inferior. The chest cavity was opened, and the lungs were filled via the trachea with Zamboni fixative (2% formaldehyde, 15% saturated picric acid in 0.1 mol/L phosphate buffer). Heart and lungs were removed en block and transferred into Zamboni fixative. After fixation for 6 h, the tissue was washed overnight with 0.1 mol/L phosphate buffer and incubated for 3 days with increasing concentrations of sucrose solution (9%, 18% and 40% sucrose in 0.1 mol/L phosphate buffer). Finally, the specimens were embedded in optimal cutting temperature (OCT) compound (Sakura; Zoeterwoude, The Netherlands) and frozen in liquid nitrogen.

### Immunohistochemistry

Immunohistochemical double-labeling of lung tissue for Ki67 (proliferating cells) and α-smooth muscle actin (vascular muscularization) was employed for a quantitative analysis of the proliferative activity of the pulmonary vasculature. For that purpose, 10 μm thick frozen sections were prepared and Ki67 antigen was unmasked by microwave treatment (twice for 6 min at 800 W in 0.1 mol/L sodium citrate buffer, pH 6.0). After blocking of unspecific protein binding sites, the frozen sections were incubated overnight simultaneously with FITC-conjugated anti-α-smooth muscle actin antibody and anti-Ki67 antibody (1:500 and 1:1000, respectively, in 5% bovine serum albumin, 5% normal goat serum in phosphate buffered saline (PBS)) followed by Cy3-conjugated donkey anti-rabbit antibody (1:2000 in 5% bovine serum albumin, 5% normal goat serum in PBS, 1 h at room temperature). After three washes with PBS the sections were incubated with 1 μg/ml DAPI in PBS for 15 min followed by three washes with PBS. Sections were evaluated with an epifluorescence microscope (BX60; Olympus, Hamburg, Germany) equipped with appropriate filter combinations. The number of cells with Ki67 positive nuclei detectable per cross section of a vessel was defined as "Ki67 positive cells/vessel". Per condition, two lung sections were analyzed, and the mean was calculated. The obtained data were statistically analyzed as described in "Statistical analysis".

The lung sections stained for α-smooth muscle actin were also used to evaluate by computer-aided planimetry the extent of muscularization of intrapulmonary vessels. For a quantitative analysis, the ratio of the number of α-smooth muscle actin positive pixels within a vessel wall and the minimal vascular diameter [μm] was calculated. Per condition about 100 vessels were analyzed and the median was calculated. The obtained data were statistically analyzed as described in "Statistical analysis".

### Assessment of right ventricular hypertrophy

Right ventricular hypertrophy was investigated employing 10 μm thick frozen sections. In detail, cross sections of the heart embracing the walls of both ventricle and the septum were prepared and routinely stained with hematoxylin-eosin, dehydrated, and embedded in Entellan (Merck, Darmstadt, Germany). Heart sections were evaluated with a BX60 microscope (Olympus, Hamburg, Germany) employing Scion VisiCapture 1.0 software (Scion Coorporation, Frederick, Maryland, USA). The ratio of right ventricular wall area to left ventricular wall area plus septum area [RV/LV+S] was used as an index of right ventricular hypertrophy. To analyze the size of individual cardiomyocytes in cross sections of the right and left ventricle wall the diameter of individual myocytes was measured using an Axioplan 2 microscope (Zeiss, Jena, Germany) and employing the AxioVision 3.0 software (Zeiss, Jena, Germany).

### Statistical analysis

Statistical analysis was performed by using SPSS Base 8.0 (SPSS Software, Munich, Germany). Percentiles 0, 25, 50, 75 and 100 are presented in box plots. Differences among experimental groups were analyzed with the Kruskal-Wallis and the Mann-Whitney tests, with p ≤ 0.05 being considered significant and p ≤ 0.01 highly significant.

## Results

### Rapamycin prevents hypoxia-induced increase of proliferative activity within the pulmonary vasculature

**Figure 1:**
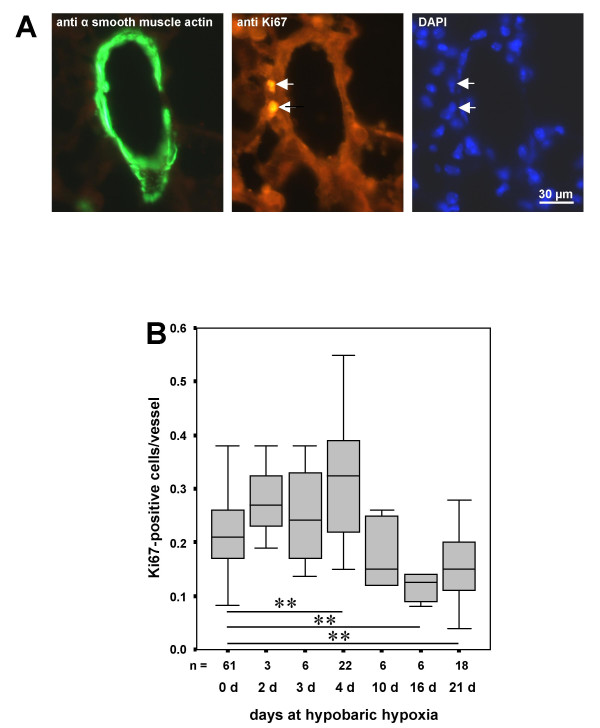
Proliferative activity in the murine pulmonary vasculature in response to hypobaric hypoxia. Frozen lung sections double immunolabeled for Ki67 and α-smooth muscle actin were used for the detection of proliferating cell within the walls of intrapulmonary vessels. Nuclei of individual cells were visualized by staining with DAPI. Exemplary immune histochemistries are demonstrated in (**A**). The results of a quantitative analysis of the number of proliferating cells/vessel depending on time of exposure to hypobaric hypoxia is given in (**B**). In the boxplots the middle horizontal line indicates the median, the top and bottom of each box identifies the upper and lower quartiles of the distribution and the top and bottom horizontal line gives the total distribution (n = number of animals. ** p ≤ 0.01).

To examine the effect of reduced oxygen supply on the kinetic of the proliferative activity within the murine pulmonary vasculature, frozen lung sections of mice housed for 0, 2, 3, 4, 10, 16, or 21 days at hypobaric hypoxia were stained for α-smooth muscle actin (smooth muscle cells) and Ki67 (proliferating cells). Nuclei of individual cells were labeled with DAPI (Fig. 1A). The quantitative analysis revealed that within the first few days hypobaric hypoxia resulted in an increased number of proliferating cells/vessel which achieved a maximum at day 4 (Fig. 1B). At that time the proliferative activity was 0.21 in normoxic mice and 0.325 in mice kept at hypoxia (p = 0.001). Thereafter, the number of proliferating cells/vessel decreased and dropped even below that seen in the normoxic control.

**Figure 2:**
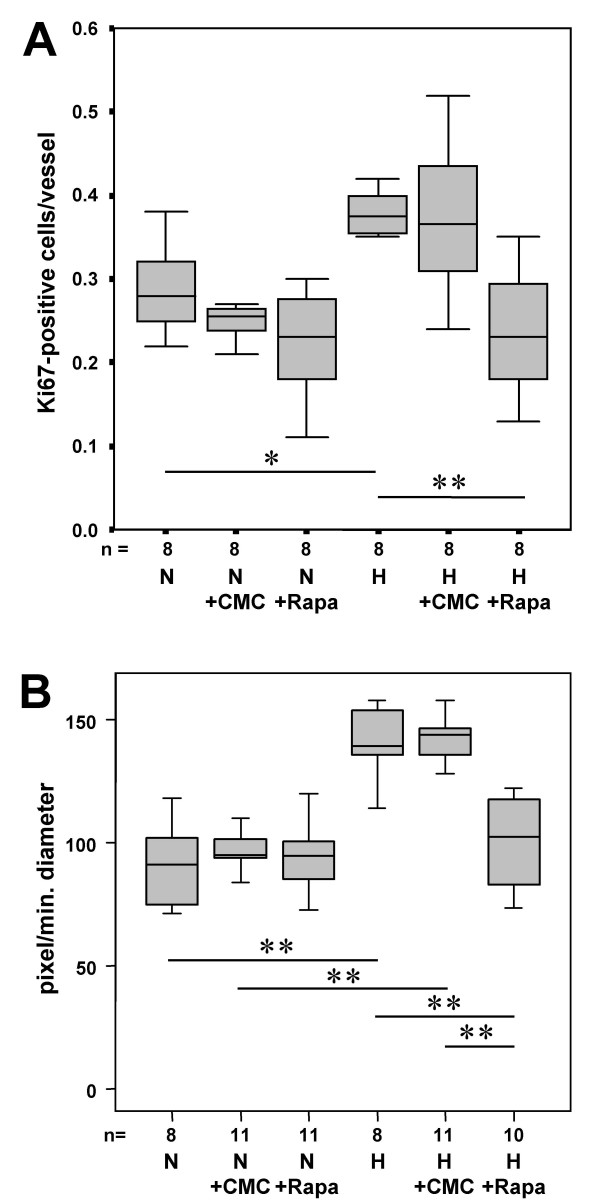
Proliferative activity and muscularization of intrapulmonary vessels of untreated mice and of animals injected with 0.2% carboxymethylcellulose as vehicle or with rapamycin. Mice were kept for four days (**A**) or three weeks (**B**) at normoxia or at hypobaric hypoxia. In frozen lung sections stained with anti-Ki67 and anti α-smooth muscle actin the number of proliferating cells per cross section of a vessel was quantified (**A**). The extent of muscularization of intrapulmonary arteries was quantified by computer-aided planimetry (**B**). The results are given as boxplots (N: normoxia; H: hypobaric hypoxia; CMC: carboxymethylcellulose; Rapa: rapamycin; n = number of animals).

Based on these results we investigated the effect of rapamycin on the proliferative activity within the pulmonary vasculature on day four of hypobaric hypoxia at which the highest proliferative activity was observed and on day 21 at which a distinct thickening of the wall of the pulmonary arteries has taken place (see below and [14]). Exposure to hypoxia for four days resulted in a significant increase in proliferative activity by 34% in untreated animals and by 43% in vehicle-injected mice (Fig. 2A). Administration of rapamycin completely abolished the hypoxia-induced increase in proliferation. The anti-proliferative effect of rapamycin was restricted to hypoxia-induced proliferation: In mice housed at normoxia the number of Ki67-positive cells/vessel was not significantly changed by rapamycin compared to the untreated (p = 0.065) and the vehicle-injected control animals, implying that rapamycin did not interfere with basal proliferative activity.

Since proliferative activity had subsided after 3 weeks of exposure to hypoxia (Fig. 1), no effect of rapamycin was detectable after this time period (data not shown).

### Rapamycin blocks the hypoxia-triggered media thickening of intrapulmonary vessels

In lung sections of mice kept for 4 days at hypobaric hypoxia a trend towards a thickened muscle layer compared to normoxic controls was already detectable. However, this difference was not significant (data not shown).

Three weeks of hypoxia, however, induced a distinct increase in muscularization of intrapulmonary vessels. The extent of muscularization rose about 53% both in untreated and vehicle-injected mice (in both cases p < 0.001) (Fig. 2B). Whereas under normoxic conditions the degree of muscularization was unchanged by rapamycin administration, in lungs of hypoxic mice a 26% reduction of the muscularization was detectable. The rapamycin-treated group did not differ significantly from animals housed at normoxia.

**Figure 3:**
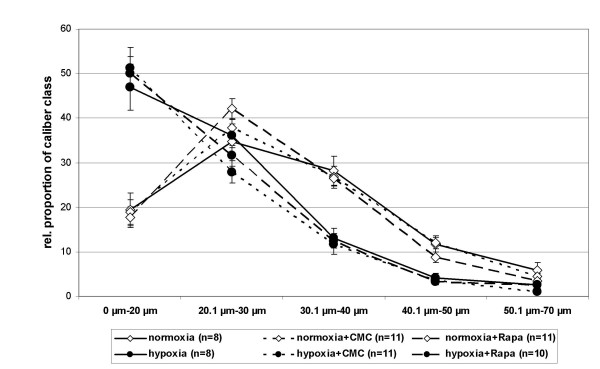
Inner diameter-based classification of intrapulmonary vessels. Three weeks of hypoxia induced a distinct shift toward smaller vessels which was not affected by CMC or rapamycin. Data are presented as means ± S.E.M (CMC: carboxymethylcellulose; Rapa: rapamycin; n = number of animals).

Allocation of the distal arteries on one of five classes of vessel caliber (inner diameter) ranging from 0 to 70 μm revealed that hypoxia-induced a distinct shift towards vessels with smaller calibers: The relative proportion of arteries with diameters smaller than 20 μm was approximately twice as high in mice kept for three weeks at hypobaric hypoxia in comparison to normobaric control animals (Fig. 3). The relative proportion of vessels with diameters between 20 and 30 μm was comparable in the normoxic and hypoxic groups. Accordingly, the relative proportion of vessel calibers of 30.1 to 40 μm as well as 40.1 to 50 μm was less in hypoxic mice. The relative proportion of vessels with large diameters (50.1–70 μm) was not different in mice housed at normoxia or hypoxia. Rapamycin treatment of mice did not affect the distribution of the vessels to the five caliber classes.

### Hypoxia-induced right ventricular wall thickening is attenuated by rapamycin

**Figure 4:**
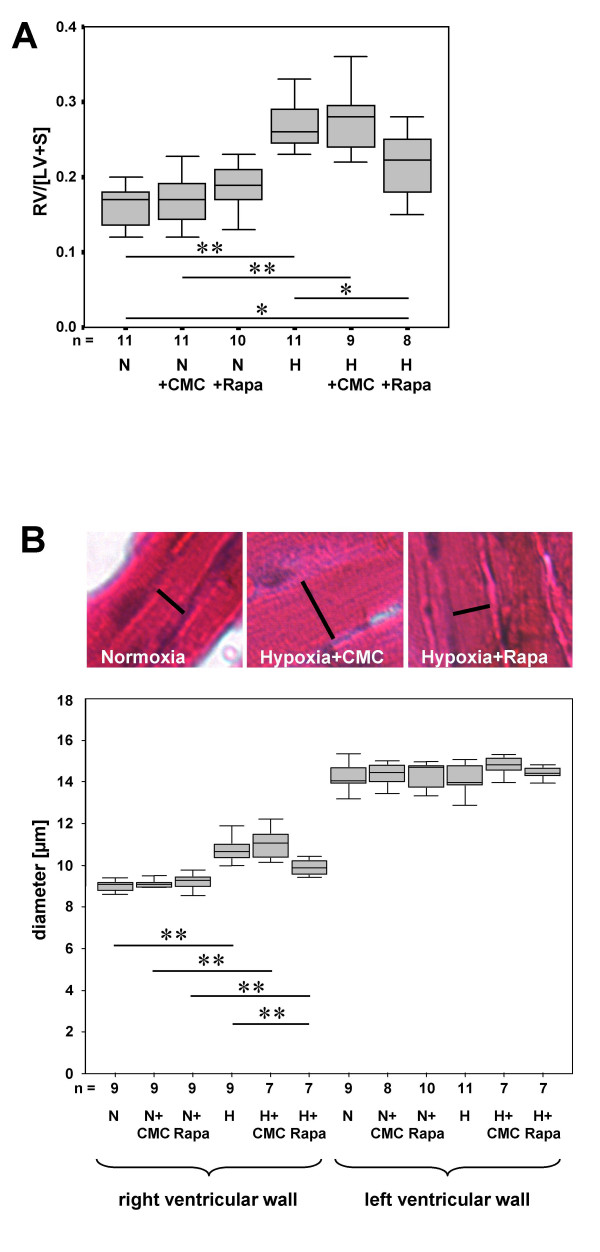
Rapamycin attenuates hypoxia-triggered thickening of the right ventricular wall and hypertrophy of individual cardiomyocytes. Hematoxylin-eosin stained frozen sections of cardiac ventricles were used to estimate the ratio of right ventricular wall area to left ventricular wall area plus septum area [RV/LV+S] (**A**). The results of a quantitative analysis of the diameters of individual cardiomyocytes of the right and left ventricular wall are given in (**B**). Data are presented as boxplots (N: normoxia; H: hypobaric hypoxia; CMC: carboxymethylcellulose; Rapa: rapamycin. n = number of animals; * p ≤ 0.05 and ** p ≤ 0.01).

Hearts of mice housed for three weeks at hypobaric hypoxia were characterized by a marked thickening of the wall of the right ventricle (Fig. 4A). The index of right ventricular hypertrophy increased about 53% and 65% in untreated or vehicle-injected mice, respectively (in both cases p < 0.001). In mice housed under conditions of reduced oxygen supply rapamycin application partially blocked the thickening of the right ventricular wall: The median was reduced by 14% compared to the untreated control group (p = 0.041) and no significant difference to vehicle- or rapamycin-injected mice kept at normoxia was detectable (p = 0.062 and p = 0.146, respectively).

### Hypoxia-triggered hypertrophy of individual cardiomyocytes is reduced by rapamycin

Untreated or vehicle-treated mice kept at hypobaric hypoxia for 3 weeks exhibited a 20% increase in cardiomyocyte diameter compared to the normoxic reference groups (p < 0.001 in both cases). Whereas rapamycin had no effect on cardiomyocyte size of mice housed at normoxia, in hypoxic animals the diameter was significantly reduced (Fig. 4B).

Cardiomyocytes of the left ventricular wall exhibited distinctly larger diameters than those of the right ventricular wall. The size of the cells was affected neither by exposure to hypobaric hypoxia nor by application of rapamycin.

### Rapamycin reverses hypoxia-induced pulmonary vascular remodeling

A therapeutic approach was probed: Mice were first exposed to hypobaric hypoxia for 3 weeks followed by another 3 weeks of hypoxia but daily rapamycin treatment. Age-matched controls were held at normoxia and treated for 3 weeks either with vehicle or with rapamycin.

**Figure 5:**
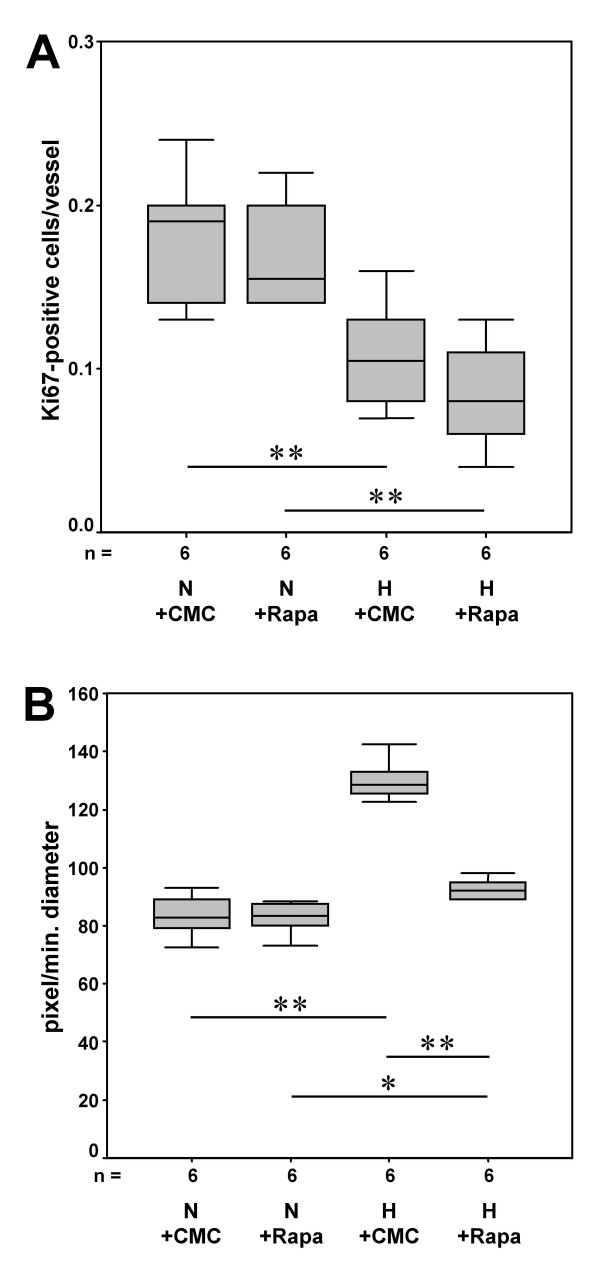
Therapeutic effect of rapamycin after induction of pulmonary vascular remodeling. Mice were exposed for three week to normoxia or hypoxia before treatment with rapamycin for three weeks. Rapamycin had no effect on proliferative activity but on muscularization of intrapulmonary vessels. Quantitative analysis of the number of proliferating cells/vessel (**A**) and of the extent of muscularization of intrapulmonary arteries as estimated by computer-aided planimetry (**B**) (N: normoxia; H: hypobaric hypoxia; CMC: carboxymethylcellulose; Rapa: rapamycin; n = number of animals; * p ≤ 0.05 and ** p ≤ 0.01)

**Figure 6:**
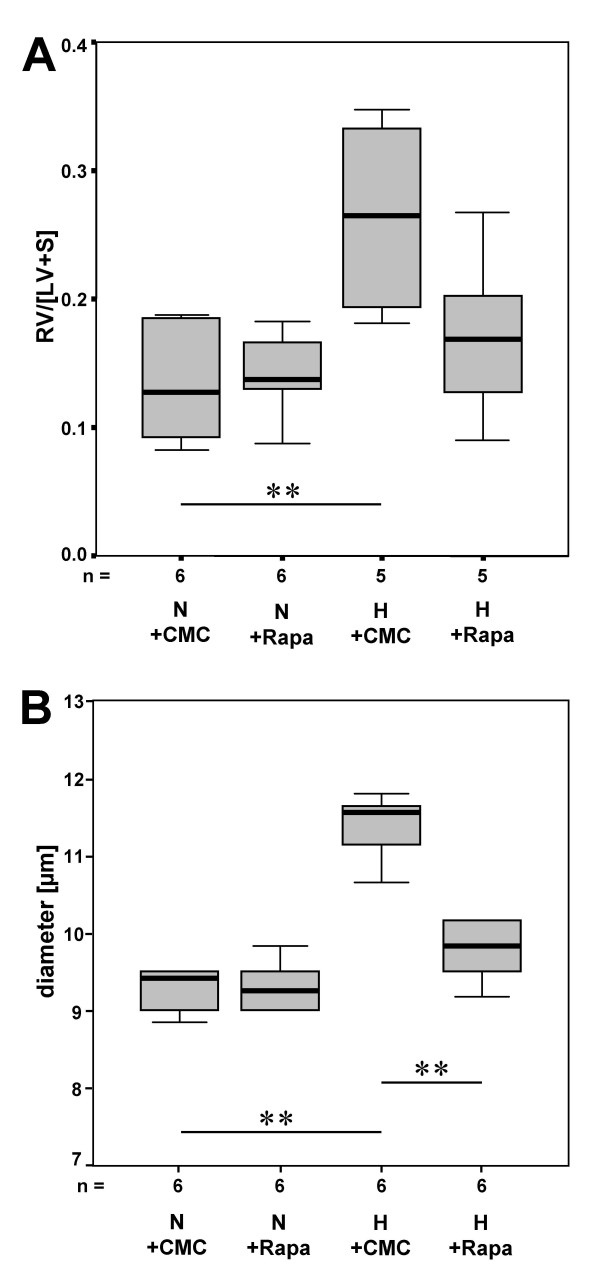
Rapamycin reverses hypoxia-induced thickening of the right ventricular wall and hypertrophy of individual cardiomyocytes. Before treatment with rapamycin mice were housed for three weeks at normoxia or hypoxia. In (**A**) the results of the estimation of the ratio of right ventricular wall/(left ventricular wall+septum) and in (**B**) a quantitative analysis of the diameters of individual cardiomyoctes are given.

In hypoxic mice proliferative activity within the vasculature was again determined even below the normoxic controls which was not further attenuated by rapamycin treatment (Fig. 5A). In contrast, 6 weeks of exposure to hypoxia had resulted in a strong 55% increase of muscularization of the pulmonary arteries (Fig. 5B). However, this increase was similar to that observed in animals kept under hypoxic conditions for only 3 weeks (see Fig. 2B) indicating that remodeling processes had reached a homeostatic situation within 3 weeks. Despite the lack of apparent proliferative activity, addition of rapamycin after 3 weeks was able to almost completely reverse vascular muscularization despite ongoing hypoxia (Fig. 5B). Accordingly, the index of right ventricular hypertrophy, which had increased twofold (208%) during hypoxia, was determined only 131% of normoxic controls when hypoxic animals were treated with rapamycin. Similarly, the increase in cardiomyocyte diameter had significantly declined (Fig. 6A and 6B).

In comparison to normoxia, hypoxia had again induced a shift of the relative proportion of arteries with diameters smaller than 20 μm. This shift was not affected by rapamycin treatment of the mice (data not shown).

## Discussion

The current medical management of PAH is directed at vasodilatation rather than towards inhibition of smooth muscle cell proliferation, although progression of pulmonary hypertension is known to be associated with increased proliferation [1]. However, the data of this experimental study imply that targeting vascular remodeling processes may represent a promising therapeutic approach towards hypoxia-induced PAH, too.

This exciting avenue has very recently been gone by Schermuly et al. demonstrating a reversal of pulmonary remodeling processes in hypoxia-induced PAH by the platelet-derived growth factor (PDGF) receptor antagonist imatinib mesylate [6]. In a case report of a patient in a desperate situation of progressing pulmonary hypertension, Seeger's group further substantiates this new concept [7]. PDGF represents a potent mitogen for pulmonary smooth muscle cells [15] acting via PI3K/Akt/mTOR, a central signaling pathway for cell cycle entry and progression. This pathway is activated by other growth factors involved in PAH as well [16] suggesting that it may represent a "final common pathway" towards proliferation. We had, therefore, successfully aimed to inhibit this pathway through usage of rapamycin which potently inhibits mTOR [8] to not only prevent but also reverse vascular remodeling processes and right ventricular signs of pulmonary hypertension in mice held under hypoxic conditions.

Hypoxia is the main stimulus for the induction of pulmonary hypertension accompanying chronic ventilatory disorders such as chronic obstructive pulmonary disease and interstitial lung disease. While acute hypoxia causes selective pulmonary arteriolar vasoconstriction, chronic exposure to hypoxia results in morphological and functional changes in the pulmonary vascular bed [17-20]. Indeed, mTOR signaling seems to play a key role in hypoxia-triggered smooth muscle and endothelial cell proliferation in vitro [12]. The requirement of PI3K, Akt, and mTOR for hypoxia-induced proliferation has also been demonstrated for pulmonary artery adventitial fibroblasts [13].

Although it is generally accepted that proliferation is an important contributor to hypoxia-induced vascular remodeling, only few data regarding the kinetics of the proliferative activity are available. Quinlan et al. [14] reported that the number of 5-bromo-2'-deoxyuridine-positive cells/vessel is about 50% higher in mice exposed to hypoxia for 4 or 6 days. After three weeks no differences in the proliferative index in the pulmonary vasculature of animals housed at normoxia or hypoxia were detectable. Our data confirm the finding of an only transient increase of proliferative activity within the pulmonary vasculature during hypoxia reaching a maximum within the first week. In our study this increase was sensitive to rapamycin treatment suggesting that inhibition of the early hypoxia-triggered cell cycle activity results in reduced chronic vascular remodeling. This way the drug may prevent further hypoxia-triggered proliferation and disease progression.

However, prevention of early proliferation does not explain rapamycin's effectiveness when given therapeutically after 3 weeks of hypoxia when proliferative activity within the pulmonary vasculature was determined even below that of normoxic mice. Rapamycin may inhibit the undetectable turnover the smooth muscle cells within the vessel wall are subjected to and, by this means, revert vascular muscularization when hypoxia had already resulted in pulmonary arterial remodeling. However, mTOR holds a critical role for activation of protein synthesis as well and, this way, seems to be involved in smooth muscle hypertrophy [21,22]. Our data, indeed, indicate that rapamycin acts as a selective inhibitor of hypoxia-induced thickening of the muscle layer: Histologically, pulmonary vascular remodeling is characterized by de novo muscularization of small precapillary vessels and by smooth muscle cell hyperplasia and hypertrophy resulting in media thickening [14,23]. With our assays we were able to quantify both processes: A classification based on the vessel caliber acted as an indicator for de novo muscularization of small arteries and the calculation of the ratio of "number of α-smooth muscle actin positive pixels within a vessel wall/minimal vascular diameter" was a criterion for the media thickening. Application of rapamycin prevented the hypoxia-induced increase in media thickening without affecting the degree of muscularization of lung vessels obtained from mice kept at normoxia. The relatively high proportion of vessels with calibers smaller than 20 μm diameter observed in mice kept at hypoxia was also detectable after rapamycin treatment. These results indicate that rapamycin had no effect on de novo muscularization of small arteries but acts as a selective inhibitor of hypoxia-induced thickening of the muscle layer. Presumably the de novo muscularization of precapillary vessels is caused by transdifferentiation of non-muscle cells and/or migration of smooth muscle cells from proximal to distal segments of the vascular system [14,23]. Although rapamycin's effect on either processes have been demonstrated in growth factor-stimulated cells [24,25], its effect on hypoxia-induced migration and/or transdifferentiation has not been investigated.

We propose that pulmonary vascular remodeling occurs in two steps: An initial phase of increased proliferative activity is followed by a second phase characterized by enhanced hypertrophy of vascular smooth muscle precursor cells leading to thickening of the media. Through its anti-proliferative and anti-hypertrophic effects rapamycin seems able to affect each phase.

An orally active derivative of rapamycin has previously been shown to attenuate monocrotaline-induced pulmonary hypertension in pneumonectomized rats [26]. In this study, the drug was only effective when started simultaneously with the administration of monocrotaline. Late therapy did not affect pulmonary hypertension. The difference to our study may be due to the different models used. Monocrotaline has been proposed to cause perivascular inflammation and platelet activation, subsequently resulting in a proliferative response of the vascular media which is augmented by increased shear stress in the pulmonary bed after pneumoctomy [27]. The difference may also lie in a transient inflammatory response after single monocrotaline treatment of rats. In this study, mice were exposed to hypoxia during the entire experimental period. Yet, this is the first study to show the effectiveness of rapamycin in preventing pulmonary hypertension induced by a pathophysiologic stimulus.

In our study, the extent of hypoxia-induced myocardial hypertrophy (RV/[LV+S]) was in good agreement with data published by other groups [28]. Additionally, the diameter of individual cardiomyocytes was determined. Hypoxia induced a 20% increase in diameters of cardiomyocytes of the right ventricular wall while cardiomyocytes of the left ventricular wall were unaffected by changes in the oxygen supply. Hypoxia-induced hypertrophy of the right ventricle and of individual right ventricular cardiomyocytes was attenuated by treatment with rapamycin. In principle, hypertrophy of the right ventricular wall in response to hypoxia is caused by the ability to respond to increased mechanical stretch caused by pulmonary vasoconstriction and vascular remodeling. However, the capability of individual cardiomyocytes to sense changes in the oxygen supply and respond with hypertrophy has been suggested, too [29]. In any case, recent data suggest that, in response to certain hypertrophic stimuli, signaling via mTOR is required for activation of protein synthesis and cardiac hypertrophy [30]. Either mechanisms may have resulted in attenuation of right ventricular hypertrophy and cardiomyocyte diameter. A direct effect of the drug on cardiomyocyte size raises the possibility of detrimental effects on right ventricular function. However, when load-induced left ventricular hypertrophy was induced in mice, rapamycin treatment resulted in a decrease in chamber size and a normal systolic function of the left ventricle [31]. However, disruption of coordinated cardiac hypertrophy has recently also been shown to contribute to the transition to heart failure as well [32].

## Conclusion

In vascular medicine, local application of rapamycin through drug-coated stent placement is currently the favored invasive strategy to prevent restenosis [33]. Systemic treatment with rapamycin is, furthermore, successfully used to prevent graft rejection and transplant vasculopathy in de novo heart transplant recipients [34]. Rapamycin may represent a novel therapeutic strategy for pulmonary arterial hypertension in humans as well to delay further progression or even result in regression of pulmonary vascular remodeling.

## Competing interests

The author(s) declare that they have no competing interests.

## Authors' contributions

RP contributed to the study design, performed histochemical analyses and drafted the manuscript.

PS performed the animal studies, morphometrical analyses, histochemical analyses and contributed to the evaluation of the data.

AvL performed histochemical and morphometrical analyses on the right heart architecture

PF and AG performed morphometrical analyses

HT contributed to the study design

WK contributed to the study design and revised the manuscript

RBD contributed to the study design, evaluated the data, and revised the manuscript
